# Novel Interleukin-11 Inhibitors Attenuate Collagen Production in Patient-Derived Synovial Fibroblasts

**DOI:** 10.7759/cureus.89850

**Published:** 2025-08-11

**Authors:** Ryan Schroeder, Miguel A De Jesus, Steven Saweikis, Molly Chaffee, Sarah Miller, Emma Richard, Cathy A Swindlehurst, Luis Marrero

**Affiliations:** 1 Orthopaedic Surgery, Louisiana State University Health Sciences Center, New Orleans, USA; 2 Research, Louisiana State University Health Sciences Center, New Orleans, USA; 3 Medicine, Louisiana State University Health Sciences Center, New Orleans, USA; 4 Biotechnology, Novomedix, San Diego, USA

**Keywords:** interleukin 11, knee arthrofibrosis, osteoarthritis (oa), synovial membrane, transforming growth factor-β1, type 1 collagen

## Abstract

Purpose: Arthrofibrosis (AF), or excessive joint scarring, is a debilitating condition that causes pain and stiffness secondary to osteoarthritis and is often worsened by the surgical trauma of total knee arthroplasty (TKA). The underlying pathology involves dysregulated transforming growth factor-beta 1 (TGF-β1) signaling, which drives fibrogenesis. However, evidence from other organ systems suggests that interleukin-11 (IL-11) mediates fibrosis downstream of TGF-β1. This study examines the relationship between IL-11 and synovial fibrosis, a hallmark of AF, in patients with end-stage knee osteoarthritis (kOA) and evaluates the potential of novel small-molecule IL-11 inhibitors (NMX compounds) as a therapeutic option.

Methods: Synovial fluid and tissue were collected from 24 patients undergoing TKA for kOA, who were divided into low (n = 12) and high (n = 12) fibrosis severity groups. Enzyme-linked immunosorbent assay (ELISA) (R&D Systems, Minneapolis, MN) and quantitative immunodetection were used to analyze IL-11 and TGF-β1 levels in synovial fluid and to relate them to histological fibrosis status. The effectiveness of NMX compounds (NM922, NM1157, and NM1332) was then tested on commercial synoviocytes and patient-derived fibroblastic synovial cells (FSCs) by measuring IL-11 and collagen type 1 (COL1) production before and during TGF-β1 stimulation in vitro. Parametric tests were employed to compare groups and evaluate associations.

Results: IL-11 and TGF-β1 levels in patient-derived synovial fluid were strongly correlated (R = 0.602; p = 0.003), and each was significantly linked to the severity of synovial fibrosis (R = 0.759; p = 0.0097 and R = 0.527; p < 0.0001, respectively). In vitro, NMX compound treatment reduced TGF-β1-induced IL-11 and COL1 by over 40% compared to untreated controls. Notably, patient-derived FSCs conserved a hyper-fibrotic phenotype, with baseline production of IL-11 and COL1 elevated by 182.86% and 139.63%, respectively, compared to commercial synoviocytes. In the pathologically primed FSCs, the lead compound NM1157 effectively countered fibrogenic stimulation, significantly reducing COL1 production by 44.5% (p = 0.0189) and IL-11 secretion by 28.4% (p = 0.0415).

Conclusions: Our findings demonstrate a strong connection between IL-11 and synovial fibrosis status in patients with advanced kOA, indicating that IL-11 is a key mediator of this process. The ability of the new NMX compounds to significantly reduce fibrotic markers in patient-derived cells offers a promising preclinical basis for developing targeted therapies to prevent or treat AF attributable to kOA or other arthropathies and traumatic joint injuries.

## Introduction

Arthrofibrosis (AF), the pathological formation of excessive scar tissue within a joint, is a common symptomatic feature of chronic arthropathy and a challenging complication following orthopedic surgery and trauma. This condition, characterized by dense intra-articular adhesions and capsular contraction, leads to debilitating pain, profound functional loss, and a significant reduction in quality of life. While frequently associated with total knee arthroplasty (TKA), where it affects up to 15% of patients [1,2], this problem is also pervasive across other orthopedic procedures. Clinically significant stiffness occurs in 2% to 35% of patients after anterior cruciate ligament (ACL) reconstruction [3] and is a common sequela of high-energy articular trauma. Conventionally, AF is addressed through a demanding regimen of physical therapy. Still, this approach is often limited by patient pain tolerance and compliance, and it is ineffective mainly once a mature, fibrotic matrix has formed [4,5]. For these refractory cases, more invasive procedures, such as manipulation under anesthesia (MUA) or surgical lysis of adhesions, are performed [2]. However, these interventions carry their risks, including fracture and instability with MUA, and a high rate of recurrence after surgical release, as the procedure itself can incite further inflammation and scarring [5,6]. Critically, the fibrotic process often begins before surgical intervention, as pre-operative stiffness is one of the strongest predictors of poor post-operative motion [7]. This clinical reality highlights a significant treatment gap: the lack of a non-invasive therapy to target the underlying biology of fibrosis, particularly in high-risk patients with pre-existing stiffness.

At its core, AF is a disease characterized by dysregulated wound healing, driven by the hyperactivation of synovial fibroblasts. A complex network of inflammatory signals converges on these cells, transforming them into proliferative, contractile myofibroblasts that aggressively deposit extracellular matrix, primarily type I collagen (COL1) [6,8-10]. Central to this pathological cascade is transforming growth factor-beta 1 (TGF-β1). This potent cytokine is widely recognized as a master regulator of tissue fibrosis [11-13]. While TGF-β1 is a logical therapeutic target, its clinical translation has been hampered by its pleiotropic nature; TGF-β1 also plays essential roles in normal tissue homeostasis and immune regulation, and its systemic inhibition carries a significant risk of adverse effects [14-16]. This therapeutic challenge highlights the urgent need to identify more specific, downstream effectors in the fibrotic pathway that can be targeted without disrupting essential biological processes.

Emerging evidence from outside of orthopedics has identified interleukin-11 (IL-11) as a critical and more specific downstream mediator of TGF-β1-induced fibrosis. In cardiac, pulmonary, and hepatic models, TGF-β1 drives the production of IL-11, which activates pro-fibrotic signaling to induce fibroblast activation and matrix deposition [17-20]. Importantly, blocking IL-11 signaling can prevent or even reverse fibrosis in these models without the broad effects of inhibiting TGF-β1 [17]. This positions IL-11 as a desirable therapeutic target. In the context of joint disease, its role is becoming clearer; IL-11 and its receptor are upregulated in the synovium of patients with osteoarthritis and rheumatoid arthritis, which are implicated in promoting inflammation and tissue degradation [21,22].

Therefore, this study was designed to bridge the clinical problem of AF with the basic science of IL-11 signaling. We first sought to establish a direct link between IL-11 levels, TGF-β1, and the histological severity of synovial fibrosis in a cohort of patients undergoing TKA for advanced osteoarthritis. We then evaluated the therapeutic potential of novel non-steroidal small-molecule compounds (NMX) that inhibit these pro-fibrotic pathways. We hypothesized that IL-11 levels would strongly correlate with fibrosis and that these NMX compounds would effectively block the production of IL-11 and COL1 in patient-derived synovial fibroblasts, providing a basis for the preclinical rationale of a novel, targeted pharmacotherapy for AF.

## Materials and methods

Patient sample collection and fibrosis analysis

With full approval from the Institutional Review Board of the Louisiana State University Health Sciences Center, New Orleans, USA (IRB# 986), synovial tissue and fluid were collected intra-operatively from 24 consenting patients undergoing primary TKA for end-stage knee osteoarthritis (kOA). A portion of each synovial tissue sample was immediately fixed in zinc-buffered formalin for histological analysis. These fixed tissues were later paraffin-embedded, sectioned, and stained with picrosirius (PS) to evaluate collagen content. The stained sections were imaged via confocal microscopy, and the total area of collagen fibrils was quantified to generate a histological fibrosis score, as previously described by our group [23]. These scores enabled the stratification of patients into two cohorts: high (n = 12) and low (n = 12) synovial fibrosis. The remainder of the fresh synovial tissue and corresponding synovial fluid were cryopreserved for subsequent cellular and molecular analyses.

Analysis of synovial fluid and tissue

Cryopreserved synovial fluid samples were thawed, cleared by centrifugation, and analyzed for IL-11 and TGF-β1 concentrations using commercially available competitive and sandwich enzyme-linked immunosorbent assay (ELISA) kits, respectively (R&D Systems, Minneapolis, MN). To confirm and localize these findings within the tissue, quantitative immunohistochemistry (qIHC) was performed on paraffin-embedded sections from 18 patient samples, limited by tissue availability. These sections were co-stained using a mouse monoclonal primary antibody for TGF-β1 and a rabbit polyclonal primary antibody for IL-11. Detection was achieved using anti-mouse Alexa 594 and anti-rabbit Alexa 647 fluorescent secondary antibodies (Jackson Immunoresearch, West Grove, PA), with 4',6-diamidino-2-phenylindole (DAPI) as a nuclear counterstain. A confocal microscope (FV1000; Olympus of America, Center Valley, PA) was used to capture images, and the background-corrected signal intensity for each protein was quantified using Slidebook™ software (3i, Denver, CO).

Cell culture and therapeutic compound treatment

We utilized two cell models to evaluate the efficacy of the novel NMX compounds: commercial human fibroblast-like synoviocytes (HFLS; Cell Applications, San Diego, CA) and patient-derived fibroblastic synovial cells (FSCs). HFLS were seeded and grown in synoviocyte growth media (SGM) to 80% confluency at 37°C and 5% CO_2_. For experiments, all cells were seeded in six-well plates, in experimental triplicate, allowed to adhere, and then serum-starved for 24 hours to synchronize them. The NMX compounds (NM922, NM1157, and NM1332), developed by Novomedix (San Diego, CA), were diluted in dimethyl sulfoxide (DMSO) and SGM to final concentrations of 0.5 µM and 2 µM. These concentrations were chosen based on prior studies that demonstrated efficacy in other fibrotic models without inducing cytotoxicity [24-26] and were evaluated using the 3-(4,5-dimethylthiazol-2-yl)-2,5-diphenyltetrazolium bromide (MTT) assay. Briefly, HFLS were seeded in 96-well plates and treated with NMX compounds at concentrations of 0.5, 1, 5, 10, and 20 µM, with n = 6 per concentration, for 48 hours, the maximum time HFLS were exposed to fibrogenic stimulation plus treatment in our experiments. Following treatment, MTT reagent was added to each well and incubated for four hours, allowing mitochondrial dehydrogenases in viable cells to convert MTT to insoluble formazan. The formazan product was then solubilized with DMSO, and absorbance was measured at 570 nm. Cell viability was calculated as a percentage relative to untreated control cells to determine dose-dependent cytotoxic effects. Two treatment protocols were employed after determining 0.5 and 2 µM as suitable, safe concentrations to measure an effect. A co-treatment protocol stimulated the cells with 4 ng/mL of recombinant TGF-β1 simultaneously with the NMX compound for 48 hours to model direct intervention. A pre-treatment protocol involved exposing cells to the NMX compound for 24 hours, followed by the addition of TGF-β1 for an additional 48 hours. This latter design investigated whether the compounds could "prime" the cells against a subsequent fibrotic stimulus, a concept with clinical relevance for pre-operative administration [27].

Fresh synovial tissues from 11 kOA patients were first minced and suspended in a cryopreservation medium consisting of 90% fetal bovine serum (FBS) and 10% dimethyl sulfoxide (DMSO) to isolate patient-derived cells. The samples underwent slow, controlled-rate freezing in a Mr. Frosty™ Freezing Container (Thermo Fisher Scientific, Waltham, MA) at -80°C and then were transferred for long-term storage into a Thermo Fisher CryoPlus™ (Thermo Fisher, Waltham, MA) vapor phase liquid nitrogen system maintained at -135°C. For experiments, the tissues were thawed and subsequently digested for 90 minutes at 37°C in RPMI 1640 Medium containing collagenase type VIII from *Clostridium histolyticum* (Sigma-Aldrich, St. Louis, MO). The resulting cell suspension was rinsed, passed through a cell strainer to remove undigested tissue, and centrifuged to pellet the cells. Patient FSCs were then cultured to 80% confluency, and viability was confirmed before they were seeded for experiments. Based on its superior performance in HFLS, only NM1157 was applied to patient FSCs at the lower concentration of 0.5 µM to assess efficacy while minimizing potential off-target effects. Triplicate wells of patient FSCs were subjected to the pre-treatment protocol as described above. For all experiments, control groups included cells stimulated with TGF-β1 alone (S/NT) and unstimulated cells cultured in SGM alone (US/NT).

Quantification of IL-11 and collagen

Following all treatment protocols, the conditioned media from the cell cultures were collected to quantify secreted IL-11 levels via competitive ELISA (R&D Systems) of technical triplicates. The remaining cells were lysed, and the protein isolate was used to quantify COL1 concentration via ELISA (Abcam, Cambridge, UK) in technical triplicates. To ensure accuracy, all measured COL1 levels were normalized to the total protein content of the cell lysate, which was determined using a bicinchoninic acid (BCA) assay (Abcam, Waltham, MA).

Statistical analysis

All data were analyzed using GraphPad Prism 10.2.3 (GraphPad Software, Inc., Boston, MA). The relationships between synovial fluid IL-11, TGF-β1, and histological fibrosis scores were evaluated using simple linear regression and Pearson’s correlation coefficient calculations. Differences in synovial fluid concentrations of cytokines, histological metrics between the high and low fibrosis cohorts, and differences in baseline IL-11 and COL-1 between HFLS and patient FSCs were assessed using an unpaired Student’s t-test. For the in vitro experiments, a two-way analysis of variance (ANOVA) was used to analyze HFLS data. A one-way ANOVA was used to analyze the patient-derived FSCs data and compare baseline values to those measured in HFLS. Dunnett’s post hoc test for multiple comparisons was applied, as fibrosis experimental groups and compound selection groups by MTT assay were compared against untreated, TGFβ1-only-stimulated cells or untreated HFLS, respectively. Alpha was set to 0.05 for all analyses.

## Results

Patient characteristics

**Figure 1:**
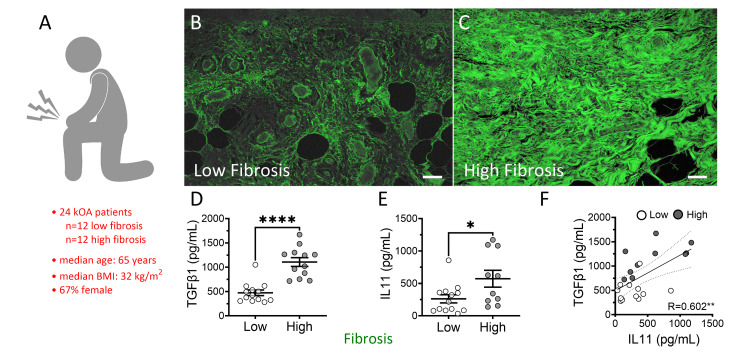
Elevated IL-11 and TGF-β1 levels correlate with high-grade synovial fibrosis in kOA patients (A) Patients undergoing TKA for kOA were categorized based on (B) low (n=12) and (C) high (n=12) histological measures of synovial fibrosis severity according to established guidelines using picrosirius (PS) fluorescence. Bars = 50 μm in representative confocal photomicrographs. (D) Synovial fluid concentrations of (D) IL-11 and (E) TGF-β1 collected from these patients during TKA were measured using competitive and sandwich ELISA, respectively, and compared as mean ± SEM with Student’s t-test (*p < 0.05 and ****p < 0.0001). (F) The levels of these two cytokines were analyzed through Pearson’s correlation, revealing a moderately high association (R = 0.6019; p = 0.0030). kOA: knee osteoarthritis, TKA: total knee arthroplasty, IL-11: interleukin-11, TGF-β1: transforming growth factor-beta 1, ELISA: enzyme-linked immunosorbent assay.

Samples were collected from 24 patients with kOA, with a median age of 65 (range 44-82) and a median BMI of 32 (range 22-58) kg/m². The cohort included 67% females (Figure 1A). Patients were classified into low and high fibrosis severity groups (Figures 1B, 1C) based on collagen fibril density measurements routinely recorded from photomicrographs of PS-stained sections of banked, formalin-fixed, paraffin-embedded synovium. Fibrosis severity was defined as low (≤ 42%) and high (≥ 54%) total collagen, as described by Hodgeson et al. [23].

Local IL-11 levels indicate synovial fibrosis status together with TGF-β1

**Figure 2:**
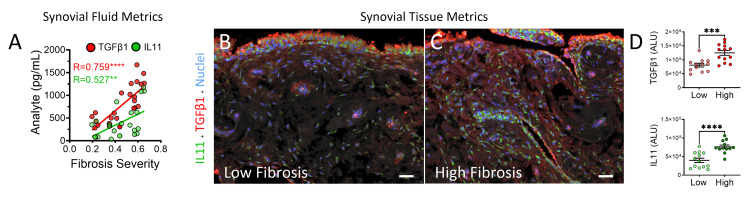
IL-11 and TGF-β1 levels in synovial fluid correlate with fibrosis severity and their distribution within synovial tissue (A) IL-11 and TGF-β1 concentrations found in synovial fluid show a high (R = 0.759; p = 0.0097) to moderate (R = 0.527; p < 0.0001) Pearson’s correlation with histological severity scores, indicating fibrosis levels. To examine these trends in tissue, IL-11 and TGF-β1 were labeled in synovial tissues collected from all patients in the (B) low and (C) high cohorts using quantitative immunohistochemistry (qIHC). Bars = 50 μm in representative confocal photomicrographs. Spatial co-distribution of (D) both cytokines was compared using Student’s t-test (***p < 0.001 and ****p < 0.0001). IL-11: interleukin-11, TGF-β1: transforming growth factor-beta 1.

TGF-β1 mean ± SEM concentrations in synovial fluid were higher in the high fibrosis group compared to the low fibrosis group (Figure 1D; 1107 ± 90 vs. 474 ± 58 pg/mL; p < 0.0001). Similarly, IL-11 was also elevated in the high fibrosis group (Figure 1E; 573 ± 130 vs. 263 ± 63 pg/mL; p = 0.0310). A moderate correlation (R = 0.60; p = 0.0030) was observed between the cytokines (Figure 1F). Individual correlations of TGF-β1 and IL-11 in the synovial fluid with histological synovial fibrosis values were also noted (Figure 2A; R = 0.76; p = 0.0097 and R = 0.53; p < 0.0001, respectively). To assess whether this trend translated into the tissue microenvironment, the distribution of the cytokines was co-detected and analyzed by qIHC in synovial tissues from patients in both fibrosis groups (Figures 2B, 2C), showing mean ± SEM signals of 76,180 ± 4,888 and 124,837 ± 8,740 arbitrary light units (ALU) for IL-11 (p < 0.0001) and TGF-β1 (p = 0.006), respectively, in the high fibrosis group compared to 39,840 ± 5,794 and 80,878 ± 6,692 ALU in the low fibrosis synovium (Figure 2D).

Selection of compound concentrations for anti-fibrogenic assays

**Figure 3:**
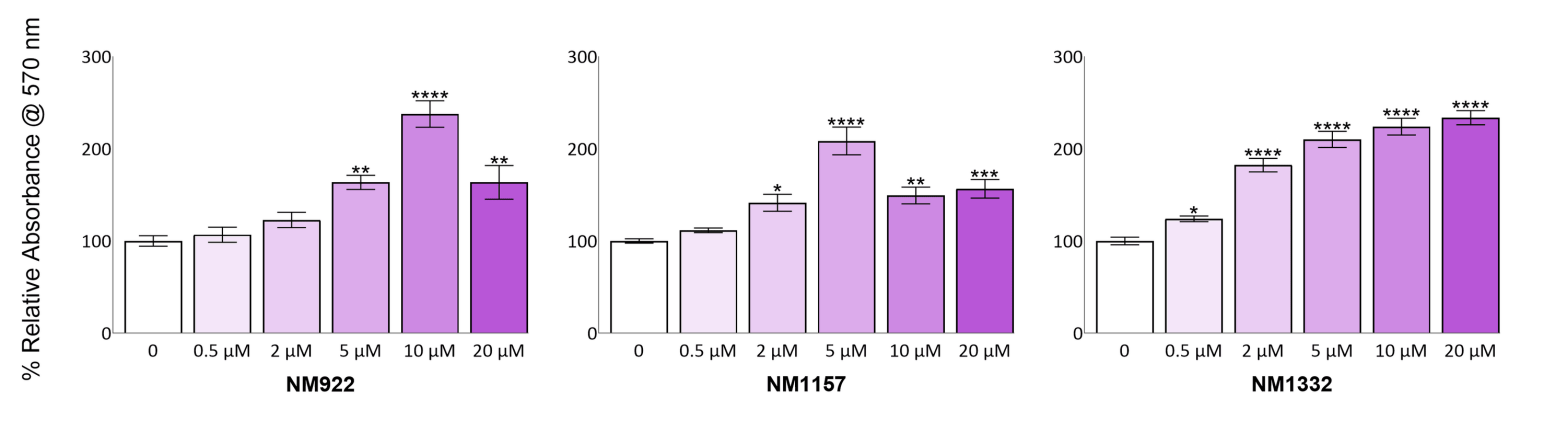
Impact of each NMX compound on HFLS metabolic activity The metabolic activity of HFLS was assessed using the MTT assay after treatment with increasing concentrations (0.5-20 µM) of NM922, NM1157, and NM1332. Data are presented as the mean percent relative absorbance compared to the untreated control (0 µM), which is set to 100%. Significance was determined using a one-way ANOVA with Dunnett's multiple comparisons test. Asterisks denote significance compared to the untreated control: *p < 0.05, **p < 0.01, ***p < 0.001, and ****p < 0.0001. HFLS: human fibroblast-like synoviocytes, MTT: 3-(4,5-dimethylthiazol-2-yl)-2,5-diphenyltetrazolium bromide.

To select appropriate compound concentrations for future anti-fibrogenic studies, we specifically sought doses that were least likely to introduce confounding alterations in the metabolic state of HFLS. Such changes, whether indicating positive effects like improved cell health and proliferation or adverse effects like oxidative stress and cytotoxicity, could hinder the straightforward interpretation of any observed anti-fibrogenic activity. Therefore, the baseline metabolic activity of HFLS was assessed across various concentrations (Figure 3). At lower concentrations, compound NM922 did not significantly differ from untreated controls at 0.5 µM (6.8% increase, p = 0.9634) or 2 µM (22.7% increase, p = 0.3235). Similarly, 0.5 µM of NM1157 did not cause a significant change (11.6% increase, p = 0.6970). Based on these results, these concentrations were deemed suitable for use in TGF-β1 stimulation experiments because they generally did not significantly deviate from the cells' baseline metabolic activity, thereby minimizing the introduction of confounding factors.

Conversely, testing at higher concentrations revealed notable increases in metabolic rate. While these increases could represent beneficial effects or efficacy in other experimental contexts, they were not aligned with our specific criterion of avoiding confounding metabolic alterations for primary anti-fibrogenic experiments. Nevertheless, these findings are an essential and beneficial consideration for future experimental designs. For example, NM1157 showed a significant effect at 2 µM (41.4% increase, p = 0.0336), with further increases at 5 µM (108.5% increase, p < 0.0001) and 20 µM (56.6% increase, p = 0.0051). Compound NM922 caused a significant increase in metabolic activity, starting at 5 µM (63.6% increase, p = 0.0031) and peaking at 10 µM (137.7% increase, p < 0.0001), followed by a decrease at 20 µM (63.6% increase, p = 0.0031). Notably, compound NM1332 was a potent inducer of metabolic activity across the entire dose range, with significant increases observed even at 0.5 µM (24.1% increase, p = 0.0343) and 2 µM (80.6% increase, p < 0.0001), continuing up to 20 µM (133.7% increase, p < 0.0001).

NMXs modulate IL-11 and COL1 in commercial synoviocytes under fibrogenic stimulus

**Figure 4:**
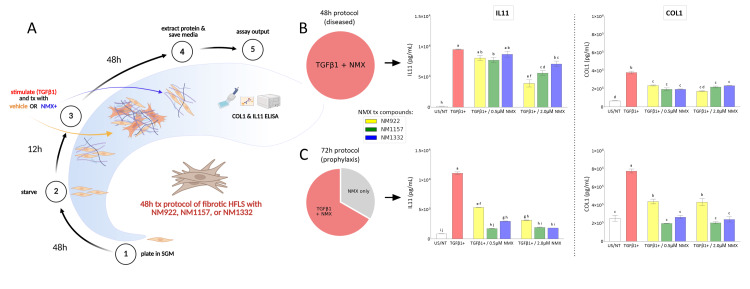
NMX compounds minimize IL-11 and collagen production in TGF-β1-stimulated, commercial synoviocytes (A) To simulate a severe diseased state, a commercial line of otherwise healthy human fibroblastic synovial cells (HFLS) was incubated with 4 ng/mL of recombinant TGF-β1 for 48 hours to induce a fibrotic phenotype while being treated with NMX compounds diluted to 0.5 and 2.0 μM. Graphic created in BioRender: Reproduced from Marrero (2025) https://BioRender.com/89a2snn [29], licensed under the Creative Commons Attribution 4.0 International License (CC-BY 4.0). To evaluate the prophylactic effect, the same protocol was applied to HFLS treated for 24 hours with NMX before 48-hour stimulation with subsequent treatment (methodology diagram not shown). At the endpoints of the (B) 48-hour and (C) 72-hour experiments (24-hour pre-treatment plus 48-hour treatment and fibrogenic stimulation), secreted IL-11 and produced COL1 were measured using competitive ELISA (with an equivalent kit to that used with the kOA patient synovial fluid) and sandwich ELISA, respectively. Data are presented as mean ± SEM, compared by two-way ANOVA. For the data shown in B and C, statistical significance was assessed using a two-way ANOVA followed by a Dunnett's multiple comparisons test, comparing each treatment group to the TGF-β1-stimulated control. In the graphs, treatment groups that do not share a letter are statistically significantly different from one another. IL-11: interleukin-11, TGF-β1: transforming growth factor-beta 1, ELISA: enzyme-linked immunosorbent assay, kOA: knee osteoarthritis, SGM: synoviocyte growth media, COL1: collagen type 1, tx: treatment, US/NT: unstimulated cells cultured in SGM alone.

To quantitatively assess the antifibrotic effectiveness of the NMX series of compounds, we induced a fibrotic phenotype in a commercial line of otherwise healthy primary HFLS. The initial experiment, a 48-hour co-treatment protocol, is illustrated schematically (Figure 4A). In this setup, HFLS were exposed to fibrogenic stimulation with a moderately high concentration of recombinant TGF-β1, which we have shown to promote this effect [28]. The test compounds were co-administered with the TGF-β1 stimulus for the full 48 hours. This co-treatment model aimed to evaluate the immediate therapeutic potential of the compounds in counteracting the effects of a fibrogenic insult on key downstream fibrosis markers, IL-11 and COL1.

In the 48-hour co-stimulation/treatment protocol, stimulation with TGF-β1 alone markedly increased the secretion of pro-fibrotic mediators, with IL-11 levels rising by 5605% (p < 0.0001) and COL1 by 454% (p < 0.0001) compared to unstimulated controls (Figure 4B). Treatment with the NMX compounds dose-dependently attenuated this response. At the 0.5 µM concentration, NM922 reduced IL-11 by 14.9% (p = 0.176) and COL1 by 37.5% (p < 0.0001); NM1157 reduced IL-11 by 18.5% (p = 0.080) and COL1 by 48.5% (p < 0.0001); and NM1332 reduced IL-11 by 8.2% (p = 0.639) and COL1 by 42.0% (p < 0.0001). At the higher 2 µM concentration, the compounds showed greater inhibition: NM922 lowered IL-11 by 58.8% (p < 0.0001)) and COL1 by 48.6% (p < 0.0001); NM1157 lowered IL-11 by 40.7% (p < 0.0014) and COL1 by 54.2% (p < 0.0001); and NM1332 lowered IL-11 by 24.9% (p = 0.021) and COL1 by 37.7% (p < 0.0001).

Next, to determine if pre-conditioning the cells enhanced therapeutic efficacy, we employed a 72-hour prophylaxis protocol that included a 24-hour pre-treatment (not shown in Figure 4A) with the compounds before the TGF-β1 stimulation protocol shown in Figure 4A. In this model (Figure 4C), TGF-β1 stimulation increased IL-11 and COL1 secretion by 281% (p < 0.0001) and 205% (p < 0.0001), respectively, over basal levels. The inhibitory effects of the compounds were notably enhanced. At 0.5 µM, NM922 reduced IL-11 by 25.4% (p < 0.0001) and COL1 by 43.3% (p < 0.0001); NM1157 reduced IL-11 by 75.2% (p < 0.0001) and COL1 by 74.3% (p < 0.0001); and NM1336 reduced IL-11 by 57.8% (p < 0.0001) and COL1 by 65.4% (p < 0.0001). At 2 µM, NM922 further reduced IL-11 by 55.6% (p < 0.0001) and COL1 by 44.0% (p < 0.0001); NM1157 reduced IL-11 by 72.4% (p < 0.0001) and COL1 by 73.5% (p < 0.0001); and NM1336 reduced IL-11 by 74.0% (p < 0.0001) and COL1 by 68.8% (p < 0.0001).

Due to its strong and consistent dose-dependent inhibitory effects across both protocols, NM1157 was identified as the leading candidate and chosen for subsequent experiments in patient-derived FSCs.

NM1157 limits IL-11 secretion and controls COL1 production in patient-derived FSCs

**Figure 5:**
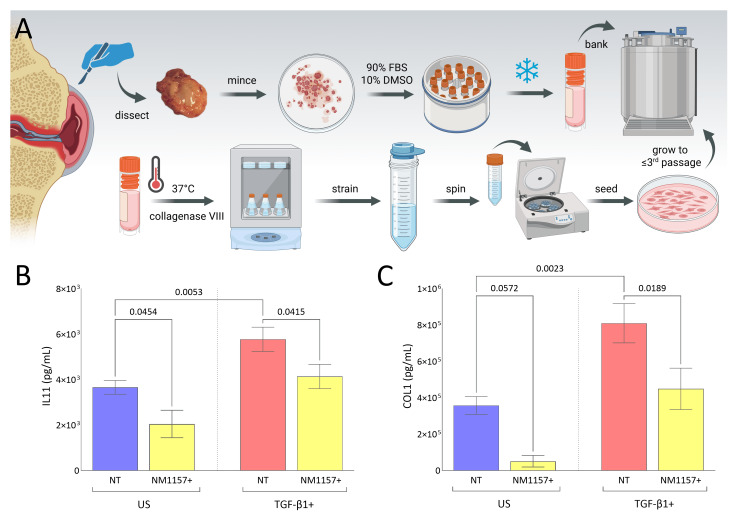
NM1157 suppresses collagen production in patient-derived fibroblastic synovial cells (A) Fibroblastic synovial cells (FSCs) derived from kOA patients with severe fibrosis were isolated from banked synovial tissue collected at the time of TKA. Graphic created in BioRender: Reproduced from Marrero (2025) https://BioRender.com/wy5d4jp [30], licensed under the Creative Commons Attribution 4.0 International License (CC-BY 4.0). Banked tissues were retrieved in batches and processed through rapid warming, dissociation, and cell purification for growth under standard culture conditions, up to the third passage. High fibrosis patient-derived FSCs (n = 12) were then plated for 48-hour NM1157 treatment at an effective dose of 0.5 µM under TGF-β1 stimulation. Similar to experimental HFLS under the same conditions, (B) IL-11 and (C) COL1 concentrations were compared among US/NT, TGF-β1 stimulated, and NM1157-treated groups under TGF-β1 stimulation. Data are presented as mean ± SEM with p-values shown and calculated by one-way ANOVA with Tukey's multiple comparisons test. kOA: knee osteoarthritis, TKA: total knee arthroplasty, TGF-β1: transforming growth factor-beta 1, HFLS: human fibroblast-like synoviocytes, IL-11: interleukin-11, COL1: collagen type 1, US/NT: unstimulated cells cultured in SGM alone, FBS: fetal bovine serum, DMSO: dimethyl sulfoxide.

To validate the therapeutic potential of NM1157 in a more clinically relevant setting, we transitioned from using commercial cell lines to an ex vivo model that utilizes FSCs derived directly from the patients with kOA included in this study. Figure 5A shows a schematic of the patient-derived cell isolation process. In brief, synovial tissue was collected during surgery from patients with kOA. The tissue was stored following standard procedures for cryopreservation to facilitate future cell culture and then processed with enzymatic digestion to release the resident cell populations. From these, primary FSCs were isolated and cultured.

Using patient-derived FSCs, we followed the 48-hour protocol (Figure 4A) used in the HFLS experiments. Mean values measured from the unstimulated, NM1157-treated groups showed a reduction in baseline IL-11 and COL1 of 44.1% (p = 0.0454) and 85.7% (p = 0.0572), respectively. Notably, there was a 182.86% (p < 0.0001) difference in IL-11 mean baseline (unstimulated) values in patient FSCs compared to US/UT HFLS. Additionally, COL1 was elevated by 139.63% (p = 0.015).

Consistent with results on HFLS, TGF-β1 stimulation strongly amplified a pro-fibrotic phenotype. Compared to unstimulated controls, TGF-β1 significantly increased IL-11 secretion by 57.7% (p = 0.0053) (Figure 5B) and markedly boosted COL1 production by 126.4% (p = 0.0023) (Figure 5C). Co-administration of TGF-β1 stimulus with 0.5 µM NM1157 effectively countered the induced fibrotic state. The compound exhibited a significant trend toward lower IL-11 levels, resulting in a 28.4% decrease in secretion compared to the TGF-β1-stimulated group (p = 0.0415). Importantly, NM1157 caused a 44.5% reduction in COL1 secretion compared to the stimulated control cells (p = 0.0189).

## Discussion

This study provides the first clinical evidence directly implicating IL-11 as a key molecular driver in the pathogenesis of synovial fibrosis in patients with kOA-attributable AF. Our data reveal a strong, linear relationship between the intra-articular concentrations of IL-11 and its upstream regulator, TGF-β1. Crucially, the levels of both cytokines correlate significantly with the histologically determined severity of synovial fibrosis, establishing a clear clinical association between this signaling axis and the pathological matrix deposition that underpins joint stiffness. This clinical link is further substantiated by our quantitative immunodetection data from fibrotic synovial tissue, which demonstrates significant spatial co-localization of TGF-β1 and IL-11 signals, cementing their functional relationship at the site of pathology.

While TGF-β1 has long been recognized as a master regulator of fibrosis, its pleiotropic nature, playing essential roles in normal tissue homeostasis, wound healing, and immune surveillance, makes it a challenging therapeutic target. Broad inhibition of TGF-β1 carries a significant risk of unpredictable and potentially detrimental off-target effects. The data presented here, however, suggest a more refined therapeutic paradigm: targeting the downstream effector, IL-11. This strategy offers the potential to selectively intercept the pathological, pro-fibrotic arm of the TGF-β1 cascade while sparing its essential homeostatic functions. This concept is strongly supported by foundational research in other organ systems; for instance, in models of cardiac fibrosis, the pro-fibrotic effects of TGF-β1 are reported to be entirely attenuated by the application of IL-11-neutralizing antibodies [19]. Furthermore, the mechanism of IL-11 action appears to involve a pathological amplification circuit where initial stimulation by TGF-β1 induces fibroblasts to produce IL-11, which then acts on the same cell or its neighbors to drive further activation and matrix production. This process can create a vicious cycle that sustains the fibrotic state, potentially becoming independent of the initial inflammatory trigger [31-33]. Therefore, the high, sustained levels of IL-11 observed in patients with severe AF are likely not just a passive response to TGF-β1 but are actively maintained by this pathological amplification loop, reframing IL-11 as a central driver of disease chronicity and a particularly compelling therapeutic target.

The therapeutic potential of the NMX compounds evaluated in this study appears to stem from a sophisticated, multi-pronged mechanism that extends beyond simple cytokine suppression. Our results in patient-derived FSCs demonstrate that NMX treatment significantly decreases COL1 deposition, the main structural component of fibrotic tissue. Notably, this strong effect on collagen was accompanied by a reduction in secreted IL-11 (p = 0.0415). This is particularly noteworthy because the patient FSCs retain a hyperfibrotic state ex vivo when compared to commercial HFLS, demonstrated by their much higher baseline IL-11 and COL1 production, in agreement with prior studies [28]. In addition to the finding that kOA patient-derived FSCs conserve a fibrotic phenotype ex vivo, already primed for elevated IL-11 secretion and COL1 production, this observation points toward a more complex mechanism of action than simple ligand suppression. A deeper mechanistic understanding, provided by preclinical data, reveals a dual-pronged strategy targeting both the pro-fibrotic "signal" and its cellular "receiver". NMX compounds function by targeting an alternative signaling complex of the Na/K ATPase (NKA) that is explicitly assembled under inflammatory and fibrotic conditions [34-36], which can amplify reactive oxygen species and is susceptible to oxidative modification [37-39]. This targeted engagement can achieve two critical outcomes: (1) silencing the downstream kinase cascades central to fibrogenesis, such as extracellular signal-regulated kinase (Erk), phosphoinositide 3-kinase (PI3K)/protein kinase B (Akt), and Janus kinase (Jak)/signal transducer and activator of transcription (STAT) pathways; and (2) promote degradation of key cell-surface receptors, including the TGF-β receptor 2 (TGFBR2) and the IL-11 receptor α (IL11Rα). This dual mechanism may help explain our findings. The profound shutdown of collagen synthesis can be achieved because either the phosphorylation of key fibrogenic kinases is limited or, even if some residual pro-fibrotic ligands, such as IL-11 or TGF-β1, remain in the microenvironment, the cell has been rendered functionally "deaf" to these stimuli by the removal of either or both receptors. This represents a far more robust anti-fibrotic strategy than ligand neutralization alone, distinguishing these compounds from less specific kinase inhibitors.

The therapeutic rationale for NMX compounds in AF is powerfully reinforced by their demonstrated efficacy in preclinical models of fibrosis affecting other organ systems, suggesting that they target a conserved, fundamental pathophysiology. In idiopathic pulmonary fibrosis (IPF) models, NMX compounds not only inhibit key signaling pathways but also reverse the established fibrotic phenotype in patient-derived lung fibroblasts and a transverse aortic constriction (TAC) myocardial fibrosis [26], an effect not seen with the standard-of-care drug pirfenidone, which is designed to modulate the lung and cardiac inflammasome [40]. In vivo, NMX treatment in a bleomycin-induced IPF model significantly reduced fibrosis, inflammation, and, critically, levels of phosphorylated STAT3 (p-STAT3), leading to the conclusion that NMX disrupts the STAT3/epithelial-mesenchymal transition (EMT) axis to restrict the myofibroblast pool. The STAT3/EMT axis is increasingly recognized as a central, druggable node in the progression of systemic fibrosis [41-43]. The evidence from cardiac fibrosis models is equally compelling and offers a glimpse into the potential for functional restoration. In a model of doxorubicin-induced cardiotoxicity, the NMX compound NM922 not only prevented the development of cardiac fibrosis but also preserved cardiac function, as measured by ejection fraction. More impressively, when administered to animals with pre-existing cardiac damage, NM922 treatment reversed the functional deficit, restoring ejection fraction reserve to normal levels. Similarly, in a transverse aortic constriction (TAC) model of pressure-overload heart failure, NMX treatment preserved left ventricular function by mitigating myocardial fibrosis [24,26].

This capacity not only to halt fibrosis but also to restore organ function is critical. Current standards of care for AF, such as aggressive physical therapy, MUA, and surgical lysis of adhesions, are reactive, often painful, and carry risks of recurrence and complications [44-46]. An unmet need remains for a pathogenesis-directed therapy that can be administered either prophylactically to prevent fibrosis or therapeutically to reverse it. The NMX platform offers the potential to fill this gap. Local, intra-articular administration of an NMX compound could supplement the standard of care by being used pre-operatively in high-risk kOA patients (e.g., those with pre-existing stiffness) to "prime" the joint against the acute injury induced by surgical trauma, or post-operatively to halt the progression of fibrosis before it becomes debilitating. This therapeutic principle extends beyond kOA and TKA to other musculoskeletal conditions plagued by fibrosis, including post-traumatic contractures following ACL reconstruction or high-energy fractures, and potentially even hypertrophic scarring and keloid formation, which share similar TGF-β1-driven pathologies. The ultimate clinical goal in AF is to prevent the formation and restore joint function by improving range of motion. It is reasonable to speculate that the exact molecular mechanisms that facilitate the functional recovery of the fibrotic heart could, within the joint, drive the remodeling of the stiff, collagen-choked capsule, leading to meaningful improvements in patient mobility.

It is essential to acknowledge the limitations of this study, which also define the path for future investigation. The investigation was conducted on cells derived from patients with chronic kOA, which serves as an excellent model of established synovial fibrosis but may differ pathologically from the acute, post-traumatic AF seen after an injury or initial surgery. Nonetheless, as pre-operative stiffness is a major predictor of post-operative outcomes, this is a highly relevant patient population. The ethical and logistical challenges of obtaining synovial tissue from healthy, age-matched individuals without joint pathology are significant. To establish a non-fibrotic baseline for our in vitro experiments, we utilized a well-characterized commercial line of HFLS, which we believe serves as a reasonable comparator for assessing the hyper-fibrotic state of the patient-derived cells. Additional limitations include the relatively small sample size and the in vitro nature of the study. The clear next step is to validate these findings in vivo. Based on this promising preclinical data, our group is advancing these compounds into animal models of post-traumatic AF to evaluate their efficacy, safety, and optimal dosing for intra-articular delivery. Future studies should also aim to delineate the specific downstream signaling pathways modulated by these inhibitors and explore the development of sustained-release formulations to maintain therapeutic concentrations within the joint space over time. Lastly, the selection of compound concentrations for our primary experiments was deliberately conservative, guided by preliminary screening to isolate anti-fibrogenic effects from confounding metabolic shifts [47]. However, the incidental observation that NMX compounds potently increase metabolic rate at higher doses is a significant finding in its own right. Pathological fibroblast-to-myofibroblast differentiation is known to involve profound metabolic reprogramming, where cells adopt a highly energetic state to sustain fibrotic activity [48,49]. The ability of NMX compounds to modulate metabolic rate suggests a potential to influence this process. Given that the NKA signaling axis is a known regulator of mitochondrial function and cellular oxidative stress [35,36,50], it is plausible that NMX compounds could help optimize metabolic health under conditions of pathological stress. This raises the intriguing possibility of therapeutically guiding the hypermetabolic, active myofibroblast back toward a more quiescent fibroblast state, a strategy that aligns with emerging anti-fibrotic therapies targeting cellular metabolism [36,51].

## Conclusions

This study establishes a strong association between the TGF-β1/IL-11 signaling axis and the severity of synovial fibrosis in kOA patients. It demonstrates that novel NMX compounds can potently suppress collagen production by patient-derived synovial cells in vitro. These findings, contextualized by compelling preclinical data from pulmonary and cardiac fibrosis models, position NMX as a promising therapeutic platform for AF. However, our results also generate critical, testable hypotheses that form the basis of our ongoing investigations. Specifically, the next phase of our research will address several key questions: First, does NMX treatment favorably modulate the matrix remodeling environment by altering the critical balance between tissue inhibitors of metalloproteinases and matrix metalloproteinases? Second, can NMX reverse a pre-established, differentiated myofibroblast phenotype in synovial cells, a feat that has proven challenging for other anti-fibrotic agents? And third, can we definitively confirm that limiting STAT3, Erk, and focal adhesion kinase (Fak) phosphorylation is the lynchpin of its anti-fibrotic action in the joint, as determined in fibrotic models such as IPF? The mechanistic understanding gained from these studies will be indispensable for translating our findings into novel, pathogenesis-directed pharmacotherapy for AF and other musculoskeletal and skin fibrotic conditions.
